# Author Correction: Untargeted high-resolution paired mass distance data mining for retrieving general chemical relationships

**DOI:** 10.1038/s42004-021-00479-1

**Published:** 2021-03-15

**Authors:** Miao Yu, Lauren Petrick

**Affiliations:** 1 Department of Environmental Medicine and Public Health, Icahn School of Medicine at Mount Sinai, New York, NY 10029 USA; 2 Institute for Exposomic Research, Icahn School of Medicine at Mount Sinai, New York, NY 10029 USA

**Keywords:** Metabolomics, Environmental chemistry, Small molecules

Correction to: *Communications Chemistry* 10.1038/s42004-020-00403-z, published online 6 November 2020.

The original version of this Article contained a number of errors in the paired mass distance matrix labeled Eq. (6). The molecular weight for FMN was incorrectly given as 424.115 Da in the mass difference between oxygen and FMN and the mass difference between FMN and reduced FMN. In addition, the exact mass values were incorrectly rounded to three decimal places rather than to a more accurate four decimal places. The paired mass distance values had been calculated using the less accurate molecular weights and have been recalculated and updated where applicable. The paired mass distance values were also inconsistently provided to three, four, or five decimal places, and these have now been consistently provided to three decimal places. These errors have been corrected in the HTML and PDF versions of the Article.

