# Supplementary material for: Untargeted high-resolution paired mass distance data mining for retrieving general chemical relationships
Source: Commun Chem. 2020 Nov 6;3:157. doi: 10.1038/s42004-020-00403-z (PMC8320691; doi:10.1038/s42004-020-00403-z)
Supplement: Supplementary file 2 — Description of Additional Supplementary Files [file 42004_2020_403_MOESM2_ESM.pdf]

Description of Additional Supplementary Files

File Name: Supplementary Data 1
Description: HMDB.csv: All of the compounds in HMDB, accessed in 2019-10-02.

File Name: Supplementary Data 2
Description: Keggrall.csv: KEGG PMDs annotation database, accessed in 2020-05-04.

File Name: Supplementary Data 3
Description: T3db.csv: All of the compounds in T3DB database, accessed in 2018-10-10.

File Name: Supplementary Data 4
Description: MTBLS28posmzrt.csv: Peaks list from MTBLS28 project.

File Name: Supplementary Data 5
Description: RSI.r: R code to reproduce all of the figures, tables and calculation in this study. The layouts of scatter points and network are relied on a random process, which might produce different figures while the topological properties should be the same.
